# Septotemporal variation in beta-adrenergic modulation of short-term dynamics in the hippocampus

**DOI:** 10.1016/j.ibneur.2021.07.002

**Published:** 2021-08-04

**Authors:** Athina Miliou, Vassilis Papaleonidopoulos, George Trompoukis, Costas Papatheodoropoulos

**Affiliations:** Laboratory of Neurophysiology, Department of Medicine, University of Patras, Rion, Greece

**Keywords:** Hippocampus, Short-term plasticity, Neuronal excitation, Noradrenaline, Beta-adrenergic receptor, Dorsal-ventral axis

## Abstract

Recent evidence shows a greater facilitating effect of beta-adrenergic receptors (β-ARs) on long-term synaptic plasticity in the ventral versus the dorsal hippocampus. Here, using field potentials from the CA1 area and a ten-pulse stimulation train of varying frequency we show that activation of β-ARs by isoproterenol preferentially facilitates the output from the dorsal hippocampus at the frequency range of 3–40 Hz without affecting short-term synaptic plasticity. Furthermore, isoproterenol increases basal synaptic transmission in the dorsal hippocampus only and enhances basal neuronal excitation more in the dorsal than the ventral hippocampus. These results suggest that β-AR-modulation of short-term neuronal dynamics differs along the longitudinal axis of the hippocampus, thereby contributing to functional specialization along the same axis.

## Highlights

• We studied the effects of isoproterenol (ISO) in dorsal (DH) and ventral (VH) hippocampus.
• ISO increased synaptic transmission and population spike more in DH than VH.
• ISO modulated short-term changes of population spike in the dorsal hippocampus only.
• ISO did not affect short-term changes of synaptic transmission in DH or VH.
• β adrenergic receptors modulate short-term changes in excitation in DH only.

## Introduction

Noradrenergic transmission is profoundly implicated in modulating several brain processes including wakening, attention, synaptic plasticity, learning/memory, and sensory processing (Berridge and Waterhouse, 2003, Aston-Jones and Cohen, 2005, Sara, 2009, Hansen and Manahan-Vaughan, 2015b, Hansen and Manahan-Vaughan, 2015a, O'Dell et al., 2015). Hippocampus, a brain structure involved in navigation, memory encoding/retrieval and processing of sensory information, among other functions (Eichenbaum et al., 2016, Goode et al., 2020), is a target region of noradrenergic system (Loy et al., 1980) and adrenergic synaptic transmission strongly modulates hippocampal long-term synaptic plasticity and hippocampus-dependent memory formation via actitation of beta-adrenergic receptors (Hansen and Manahan-Vaughan, 2015b, Hansen and Manahan-Vaughan, 2015a, O'Dell et al., 2015, Hagena et al., 2016). Importantly, adrenergic transmission differs along the longitudinal (septotemporal or dorsoventral) axis of the hippocampus in terms of density of adrenergic nerve terminals (Gage and Thompson, 1980), noradrenaline content (Loy et al., 1980), extracellular levels of noradrenaline (Haring and Davis, 1985, Hortnagl et al., 1991) and modulation of long-term synaptic plasticity (Papaleonidopoulos and Papatheodoropoulos, 2018), which have all been found to be higher in the ventral compared with the dorsal hippocampus. These differences belong to a steadily growing body of evidence for intrinsic specializations of anatomical and functional organization along the hippocampal septotemporal axis that corroborates the concept of segregation of functions along the same axis (van Strien et al., 2009, Fanselow and Dong, 2010, Bannerman et al., 2014, Strange et al., 2014).

Frequency-dependent transient changes in neuronal activity, expressed as short-term changes in synaptic input and neuronal output are deeply implicated in neural information processing, including temporal filtering, synaptic input diversification and dynamic gain control (Abbott and Regehr, 2004, Jackman and Regehr, 2017). Short-term changes of neuronal input and output hereafter alternatively referred to as the short-term synaptic plasticity and short-term dynamics of neuronal excitation, respectively, are related but distinct phenomena. For instance, short-term dynamics of neuronal excitation are to some extent influenced by short-term synaptic plasticity, however, they may be determined by additional mechanisms, including synaptic inhibition (Haider and McCormick, 2009, Womelsdorf et al., 2014, Koutsoumpa and Papatheodoropoulos, 2019).

Neuromodulators play crucial roles in controlling neural information flow in brain circuits in a frequency-dependent manner (Ito and Schuman, 2008). Recently, it has been reported that short-term neuronal dynamics greatly differ along the longitudinal hippocampal axis (Papaleonidopoulos et al., 2017, Koutsoumpa and Papatheodoropoulos, 2019). Considering the greater facilitating effect of β-ARs on long-term synaptic plasticity in the ventral versus the dorsal hippocampus (Papaleonidopoulos and Papatheodoropoulos, 2018), we hypothesized that activation of β-ARs might also modulate short-term changes in neuronal activity more in the ventral than the dorsal hippocampus. To address this hypothesis, we recorded field potentials from somatic and apical dendritic layers of the CA1 hippocampal field following application of a 10-pulse stimulation train of varying frequency (0.1–100 Hz) at Schaffer collaterals. Strikingly, we found that activation of β-ARs by isoproterenol (1 μM) significantly modulates short-term dynamics of the CA1 hippocampal output in the dorsal but not the ventral hippocampus without affecting short-term synaptic plasticity in either segment of the hippocampus. Also, isoproterenol increases basal transmission and network excitation more in the dorsal than the ventral hippocampus. These results show that short-term dynamics of local CA1 hippocampal network are differently modulated by β-ARs in the two segments of the hippocampus.

## Materials and methods

### Preparation of hippocampal slices

**Fig. 1:**
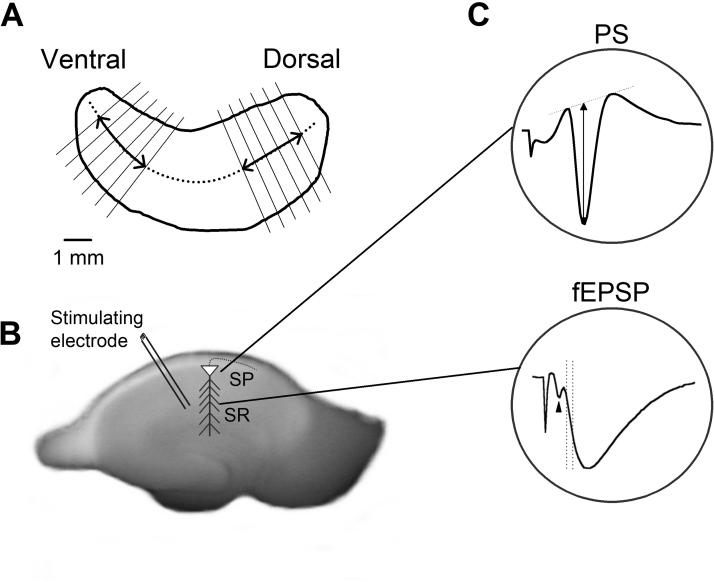
Methods used for preparing hippocampal slices and recording and quantifying field potentials. A. Drawing of a left hippocampus showing the cutting blade orientation (solid thin lines) used for preparing transverse slices (500 µm); we cut hippocampus orthogonally to its long axis (curved dotted line). Slices were prepared from the dorsal and the ventral segment of the hippocampus, extending between 1.0 and 3.5 mm from each end of the structure (solid lines with arrowheads). B. Photograph of a hippocampal slice showing the positions of stimulating and recording electrodes. Stimulating electrode was placed in CA1 stratum radiatum (SR) to activate Schaffer collaterals and recording electrodes were positioned in stratum radiatum and stratum pyramidale (SP) to record fEPSP and PS, respectively. C. The fEPSP was quantified by the maximum slope of its initial rising phase measured in a time frame of about one millisecond (denoted by the two vertical dashed lines), starting one millisecond after the peak of the presynaptic fiber volley (arrowhead). The PS was quantified by its amplitude, measured as the length of the projection of the negative peak (vertical line with arrowheads) on the line joining the two positive peaks on either side of the negative peak of the waveform (oblique dashed line).

Transverse 500 µm-thick hippocampal slices were prepared from the dorsal and the ventral segment of the hippocampi obtained from adult (3–4 months old) male Wistar rats, as previously described (Papaleonidopoulos and Papatheodoropoulos, 2018). Experiments were conducted in accordance with the European Communities Council Directive Guidelines for the care and use of Laboratory animals (2010/63/EU – European Commission) and approved by the “Protocol Evaluation Committee” of the Department of Medicine of the University of Patras and the Directorate of Veterinary Services of the Achaia Prefecture of Western Greece Region (reg. number: 187531/626, 26/06/2018). Furthermore, all efforts were made to minimize the number of animals used as well as their suffering. Following decapitation under deep anesthesia, the brain was removed, placed in ice-cold (2–4 °C) standard medium containing, in mM: 124 NaCl, 4 KCl, 2 CaCl_2,_ 2 MgSO_4,_ 26 NaHCO_3_, 1.25 NaH_2_PO_4_ and 10 glucose and equilibrated with 95% O_2_ and 5% CO_2_ gas mixture at a pH= 7.4. Each hippocampus was excised free from the brain and transverse slices, 500 µm-thick, were prepared from the dorsal (septal) and the ventral (temporal) segment of the hippocampus, as shown in Fig. 1A. Slices were immediately transferred to an interface type recording chamber continuously perfused with standard medium, of the same composition as described above, at a rate of ~1.5 ml/min. Slices were continuously humidified with a mixed gas consisting of 95% O_2_ and 5% CO_2_ at a constant temperature of 30 ± 0.5 °C. Tissue stimulation and recording started at least one and a half hours after their placement in the chamber. We used the β-AR agonist (+)-isoproterenol (+)-bitartrate salt (isoproterenol, 1 μΜ) and the β-AR antagonist (±)-propranolol hydrochloride (propranolol, 10 μΜ); both substances were purchased from Sigma-Aldrich (Germany).

### Recordings and data analysis

We recorded evoked field excitatory postsynaptic potentials (fEPSPs) from the stratum radiatum and population spikes (PS) from the stratum pyramidale of CA1 region (Fig. 1B-C) using a 7 µm-thick carbon fiber-made electrode (Kation Scientific, Minneapolis, USA). Field potentials were evoked by electrical stimulation of Schaffer collaterals using a home-made bipolar wire electrode (25 µm diameter) with an inter-wire distance of 100 µm; we used a platinum/iridium wire purchased from World Precision Instruments, USA. Baseline stimulation was delivered every 30 s. Stimulation and recording electrodes were positioned in the middle of the stratum radiatum and stratum pyramidale both in the transverse and the radial axis, using a stereo microscope (Olympus, Japan). Specifically, to stimulate afferent fibers and record fEPSP, electrodes were positioned at a distance from the pyramidal layer of 250 µm in dorsal and 300–350 µm in ventral hippocampal slices, considering that the length of a CA1 pyramidal cell is about 25–30% higher in the ventral than the dorsal hippocampus (Dougherty et al., 2012). Furthermore, stratum radiatum was determined by the negativity of the largest amplitude produced after stimulation of Schaffer collaterals at the same radial level, while stratum pyramidale was determined by the appearance of a waveform with clearly detected positivities on either side of the sharp negativity. Signal was amplified 500 times and band-pass filtered at 0.5 Hz–2 kHz using Neurolog amplifiers (Digitimer Limited, UK), digitized at 10 kHz and stored on a computer disk for off-line analysis using the CED 1401-plus interface and the Signal6 software (Cambridge Electronic Design, Cambridge, UK).

Short-term changes in fEPSP and PS were studied using a frequency stimulation protocol consisted of a ten-pulses train delivered at a frequency range from 0.1 to 100 Hz. Consecutive stimulation trains were delivered at a random fashion regarding stimulation frequency, and trains were separated by a two-minute interval. Frequency stimulation was given before and during application of the agonists of β-ARs isoproterenol (1 μM). fEPSP was quantified by the maximum slope of its initial rising phase and PS was quantified by its amplitude, as shown in Fig. 1C. The effects of frequency stimulation were quantified as the percent change of each of the nine consecutive evoked responses with respect to the first response in a train. Steady state response was estimated by averaging the responses evoked by the last three pulses in a train (i.e. 8th-10th). The parametric paired and independent *t*-tests, and the univariate full factorial general linear model (UNIANOVA) were used. The values in the text and figures express mean ± S.E.M. The number of slices and animals used is given throughout the text (slices/animals). The statistics were performed using the number of slices. The IBM SPSS Statistics 27 software package was used for all statistical analyses.

## Results

**Fig. 2:**
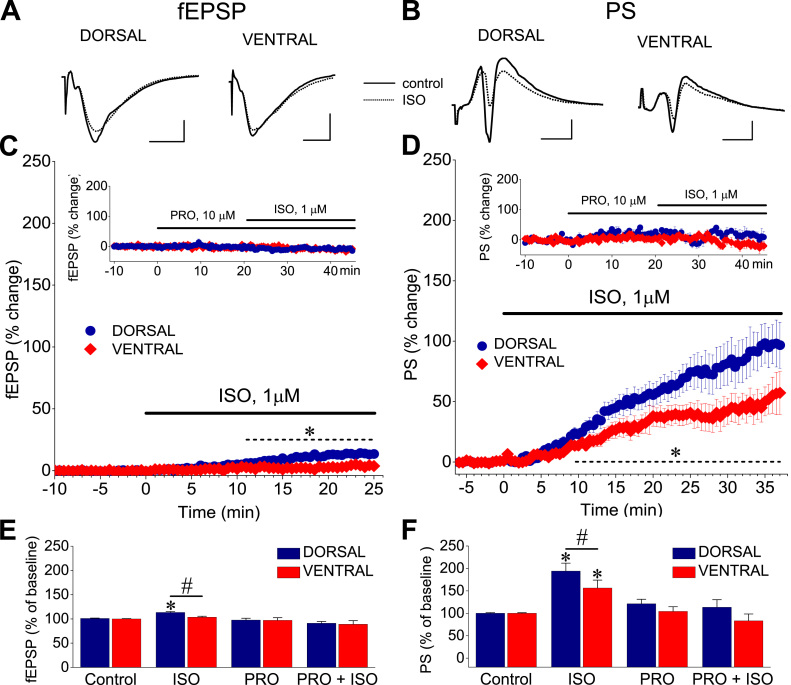
Isoproterenol increases fEPSP and PS significantly more in the dorsal than in the ventral hippocampus. A-B. Representative fEPSP (A) and PS traces (B) before (control, dotted lines) and during application of isoproterenol (ISO, solid lines) are shown. Calibration bars: 1 mV, 5 ms. Stimulation artifacts are truncated for clarity. C-D. Collective time courses of the effect of isoproterenol (ISO) on fEPSP and PS. Insert graphs show that the effects of isoproterenol were occluded by prior treatment with 10 μM propranolol (PRO). E-F. Overall results of drug action on fEPSP and PS. Asterisks denote significant drug effect (paired *t*-test, at p < 0.05), and dieses indicate significant dorsal-ventral differences (independent *t*-test, at p < 0.05).

Application of the β-AR agonist isoproterenol (1 μM), produced a significant increase in fEPSP in the dorsal but not the ventral hippocampus (Fig. 2A, C & E), and significantly enhanced PS in both segments of the hippocampus (Fig. 2B, D & F). In addition, these actions were significantly higher in the dorsal compared with the ventral hippocampus (Fig. 2E & F). Specifically, isoproterenol significantly enhanced fEPSP in the dorsal (by 13.3 ± 1.92%, n = 19/7, p < 0.005) but not the ventral hippocampus (3.59 ± 1.87%, n = 14/6, p > 0.05) (dorsal-ventral difference, p < 0.05), and increased PS more in the dorsal (by 94.15 ± 17.63%, n = 49/25, p < 0.001) than the ventral hippocampus (by 56.32 ± 17.64%, n = 40/23, p < 0.001), (dorsal-ventral difference, p < 0.05). The effects of isoproterenol were occluded by pretreatment with 10 μM propranolol (Fig. 2C & D, insert graphs). Specifically, application of isoproterenol in the presence of propranolol did not significanty change fEPSP and PS in the dorsal (n = 5/3, paired *t*-test, p > 0.05) and the ventral hippocampus (n = 5/3, paired *t*-test, p > 0.05). Also, application of propranolol did not significanty affect either fEPSP or PS in the dorsal (n = 5/3, paired *t*-test, p > 0.05) and the ventral hippocampus (n = 5/3, paired *t*-test, p > 0.05).

**Fig. 3:**
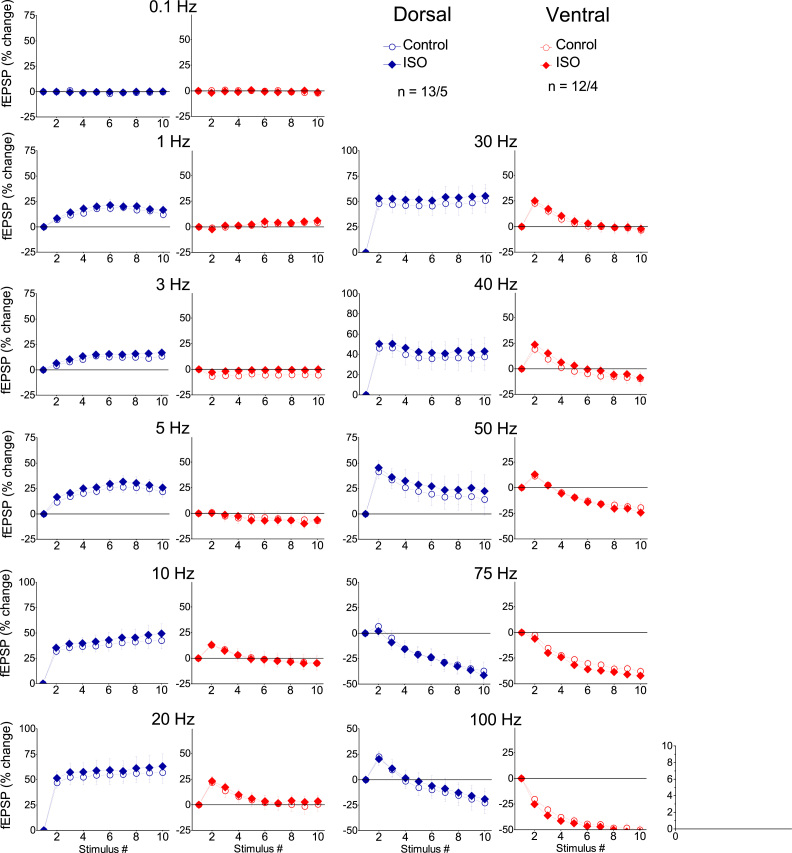
Isoproterenol does not significantly affect short-term synaptic plasticity in either segment of the hippocampus. Short-term synaptic plasticity was studied at a stimulation current intensity producing a subthreshold fEPSP slope of about 1 mV/ms. Changes in fEPSP are plotted as a function of stimulus number. Each graph shows fEPSP changes induced by a single stimulation frequency. Graphs for the dorsal and the ventral hippocampus are displayed in left and right panels, respectively.

**Fig. 4:**
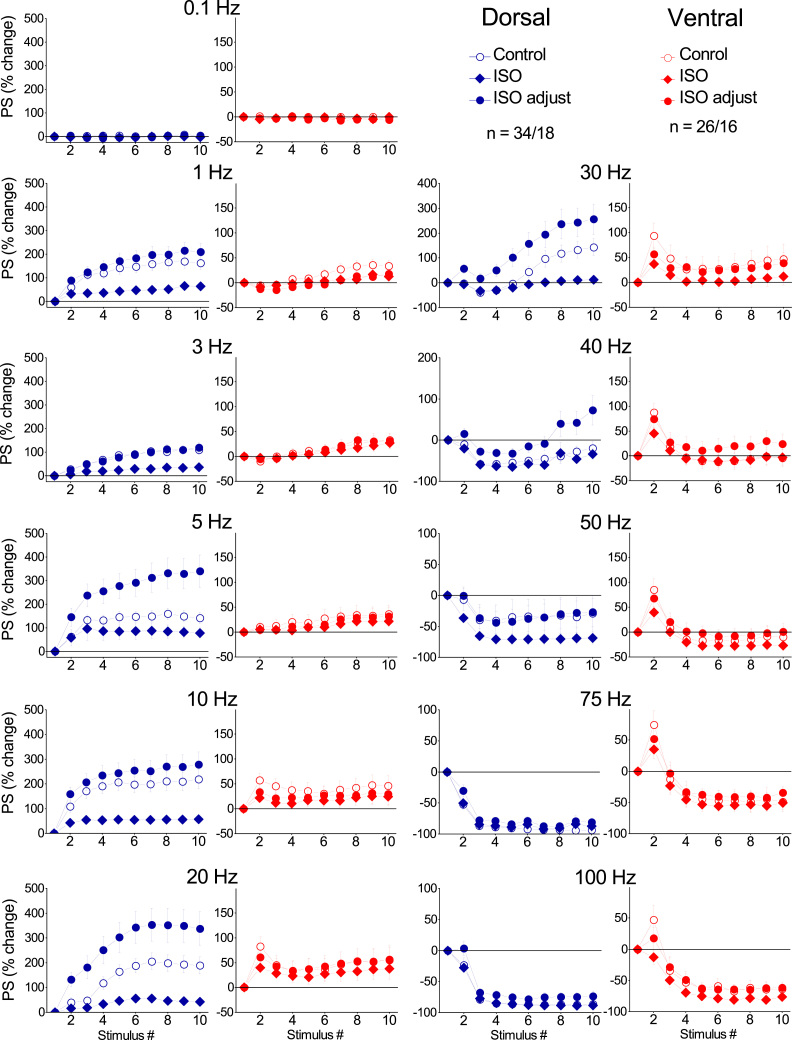
Isoproterenol significantly influences short-term dynamics of hippocampal output (PS) in the dorsal but not the ventral hippocampus. Short-term changes in PS were studied at a stimulation current intensity producing a PS of about 1 mV. Changes in PS are plotted as a function of stimulus number. The indication "ISO adjust" refers to the data collected under isoproterenol after adjusting conditioning PS to control levels. Graphs for the dorsal and the ventral hippocampus are displayed in left and right panels, respectively.

**Fig. 5:**
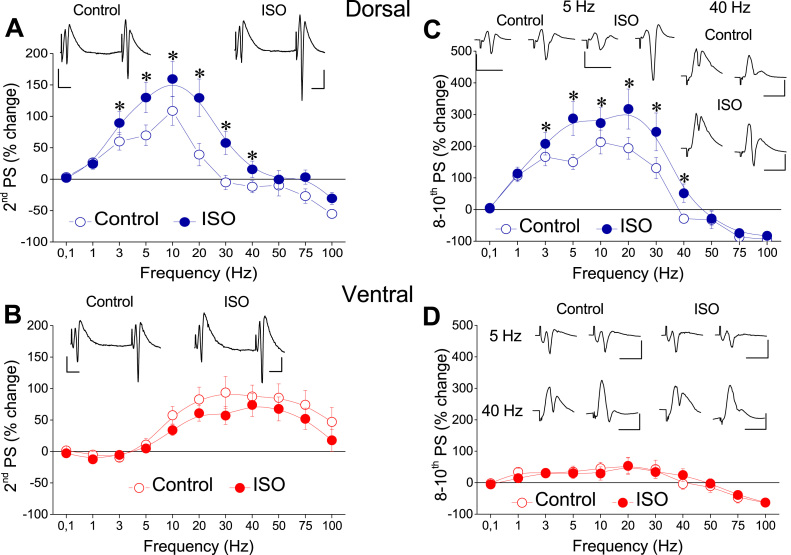
Effects of isoproterenol on conditioned responses in the dorsal and the ventral hippocampus. Data are shown for the 2nd response (A & B) and the steady-state response (C & D) in a train. In drug condition, only data with adjusted PS are shown. Example traces are given in inserts (stimulation artifacts are truncated for clarity). In plots A and B only the first two responses induced in a 20 Hz train are shown to illustrate changes in paired-pulse facilitation. In plots C and D only the first and tenth (i.e. last) responses are shown to illustrate the change induced in steady-state responses by 5 Hz and 40 Hz stimulation. Calibration bars: 0.5 mV and 10 ms in A and B, 1 mV and 10 ms in C and D. All stimulation artifacts are truncated for clarity. Asterisks indicate statistically significant drug-induced differences at *p* < 0.05 (independent *t*-test). Note that isoproterenol significantly enhances paired-pulse and steady-state facilitation of PS in the dorsal hippocampus only.

Applying the frequency stimulation protocol, we found robust frequency-dependent changes in both fEPSP (Fig. 3) and PS (Fig. 4), which greatly differ between the dorsal and the ventral hippocampus as described previously (Papaleonidopoulos et al., 2017, Koutsoumpa and Papatheodoropoulos, 2019). Furthermore, Fig. 3 shows that application of frequency stimulation under conditions of tissue perfusion with 1 μM isoproterenol did not significantly affect short-term changes in fEPSP in either the dorsal (n = 13/5, UNIANOVA, average of all conditioned responses, *F*=0.055, *p* > 0.5, second response, *F*=0.213, *p* > 0.5, steady-state response, *F*=0.046, *p* > 0.5; paired *t*-test, each response at individual frequencies, *p* > 0.05) or the ventral hippocampus (n = 12/4, UNIANOVA, average of all conditioned responses, *F*=0.227 *p* > 0.5, second response, *F*=0.357, steady-state response, *F*=0.179, *p* > 0.5; paired *t*-test, each response at individual frequencies, p > 0.05). However, application of isoproterenol in the dorsal hippocampus strongly modulated short-term dynamics of neuronal excitation (n = 34/18, UNIANOVA, average of all conditioned responses, *F*=6.404, *p* < 0.001, steady-state response, *F*=6.651, *p* < 0.001), (Fig. 4). More specifically, isoproterenol produced a significant reduction in the facilitation of steady-state responses at stimulation frequencies of 1–30 Hz (paired *t*-test at individual frequencies, *p* < 0.005). Given, however, that isoproterenol produced a robust increase of PS in both segments of the hippocampus and considering that the level of neuronal activation significantly determines short-term dynamics of excitation (Koutsoumpa and Papatheodoropoulos, 2019), the above described effects of isoproterenol on short-term changes of PS may be secondary to drug-induced increase in PS. Therefore, we examined short-term changes of PS also after adjusting the amplitude of conditioning PS (i.e., the first PS evoked by a stimulation train) to control levels (Fig. 4, ISO adjust). Remarkably, we found that in the dorsal hippocampus isoproterenol significantly modulated short-term changes of PS across most of the stimulation frequencies (UNIANOVA, average of all conditioned responses, *F*=2.268, *p* < 0.05, second response, *F*=1.869, *p* < 0.05, steady-state response, *F*=2.057, *p* < 0.05). More specifically, we found that isoproterenol significantly increased frequency facilitation at stimulation frequencies between 3 and 30 Hz and converted frequency depression into facilitation at the stimulation frequency of 40 Hz (Fig. 5A & Fig. 5C, respectively; paired *t*-test at individual frequencies, *p* < 0.05). Strikingly, in the ventral hippocampus (Fig. 4, Ventral), isoproterenol did not significantly affect short-term changes of neuronal excitation under conditions of adjusted PS (n = 26/16, UNIANOVA, average of all conditioned responses, *F*=0.209, *p* > 0.5, second response, *F*=0.294, *p* > 0. 5, steady-state response, *F*=0.283, *p* > 0.5). Similarly, under conditions of non-adjusted PS, isoproterenol did not significantly affect the average of all conditioned responses (UNIANOVA, *F*=0.401, *p* > 0.5) or steady-state responses (UNIANOVA, *F*=0.308, *p* > 0.5); yet, it significantly reduced the facilitation of the second response in a train (UNIANOVA, *F*=3.405, *p* < 0.001). Fig. 5 shows the effects of isoproterenol on the second (panels A & B) and steady-state responses (panels C & D) in the dorsal and the ventral segments of the hippocampus across all tested stimulation frequencies, under conditions of PS adjustment to control levels. Note that isoproterenol modulated short-term changes of PS in the dorsal but not the ventral hippocampus.

## Discussion

Short-term dynamics of neuronal activity profoundly regulate neural information flow in local circuits (Abbott and Regehr, 2004, Jackman and Regehr, 2017, Pariz et al., 2018) and neuromodulation represents a basic mechanism that determines or shapes information transfer between neural elements (Ito and Schuman, 2008). Importantly, neuromodulatory actions of several neuromodulators may considerably differ along the septotemporal axis of the hippocampus, see examples in Malik and Johnston, 2017, Dubovyk and Manahan-Vaughan, 2018, Mlinar and Corradetti, 2018, Papaleonidopoulos et al., 2018), and have significant implications in hippocampal physiology and pathology, discussed in Gulyaeva (2019). Neuromodulation is one aspect of the diversification in functional organization along the longitudinal axis of the hippocampus. The concept of functional segregation has emerged from research performed during the last few decades and shows that distinct segments along the hippocampus are involved to different degrees in hippocampal functions. Initially, the concept of functional segregation emerged as a difference in the involvement of the dorsal and the ventral hippocampus to spatial learning and memory, for a review see Moser and Moser (1998), while later was expressed by the dichotomy between cognition and emotionality that have been ascribed to the dorsal and the ventral hippocampus, respectively (van Strien et al., 2009, Fanselow and Dong, 2010, Bannerman et al., 2014, Strange et al., 2014). More specifically, the dorsal hippocampus has been linked to information processing underlying spatial learning and memory (Moser et al., 1993, Jung et al., 1994, Maurer et al., 2005), while the ventral hippocampus has been associated with anxiety-related behaviors (Bannerman et al., 2002, Kjelstrup et al., 2002, Pentkowski et al., 2006), social interactions, stress-induced disfunctions (McHugh et al., 2004, Okuyama et al., 2016), but also with positive emotions (Yang and Wang, 2017).

Noradrenergic transmission is a powerful modulator of neuronal activity and activity-dependent plasticity in the hippocampus (Segal and Bloom, 1974, Madison and Nicoll, 1982, Hopkins and Johnston, 1984, Dunwiddie et al., 1992, Hansen and Manahan-Vaughan, 2015b, Hansen and Manahan-Vaughan, 2015a); for some recent reviews see O'Dell et al., 2015, Hagena et al., 2016). In the hippocampus, β-ARs located at both presynaptic and postsynaptic sites regulate transmitter release and neuronal excitability (Dunwiddie et al., 1992, Gereau and Conn, 1994b, Gereau and Conn, 1994a, Pedarzani and Storm, 1996, Hoffman and Johnston, 1999, Milner et al., 2000); for a recent review see Hagena et al. (2016) Consistently, we found that isoproterenol augments transmission and neuronal excitation in CA1 field, but to a different extent in the dorsal and ventral hippocampus. In addition, we found that isoproterenol enhances short-term changes in neuronal excitation in the dorsal hippocampus only. The increased effects of isoproterenol in the dorsal hippocampus may be related to the higher expression of β1 adrenergic receptors in the dorsal compared with the ventral hippocampus, reported recently (Grigoryan and Segal, 2015), and/or a different functional coupling between β-ARs and downstream processes. It has been previously shown that β-ARs enhance excitability in CA1 pyramidal cells (Madison and Nicoll, 1982, Hoffman and Johnston, 1999) by inhibiting different types of potassium channels (Pedarzani and Storm, 1993, Hoffman and Johnston, 1999, Liu et al., 2017, Church et al., 2019) and suppressing medium afterhyperpolarization (Church et al., 2019). More specifically, β-ARs increase neuronal excitability by inhibiting Kv4.2/A-type (Yuan et al., 2002) and Kir potassium channels (Roy and Sontheimer, 1995), which are both expressed more in the dorsal than the ventral hippocampus (Marcelin et al., 2012, Kim and Johnston, 2015, Malik and Johnston, 2017). Furthermore, the β-AR-induced increase in excitation of the dorsal hippocampus occurs at a frequency range (3–40 Hz) that corresponds to the time-window of medium afterhyperpolarization (50–200 ms), (Gu et al., 2005) which regulates CA1 cell firing and involves activation of Kv7 (KCNQ) potassium channels, which are expressed more in dorsal than in ventral hippocampus (Honigsperger et al., 2015, Trompoukis et al., 2020).

Here we show that 1 μM isoproterenol modulates short-term changes in neuronal excitation in the dorsal, not ventral hippocampus, though it does not affect short-term synaptic plasticity in either segment of the hippocampus. This effect appears to be one among many facets of β-AR-dependent modulation of neuronal activity along the hippocampus. For instance, activation of β-ARs by 1 μM isoproterenol facilitates long-term synaptic potentiation (LTP) induced by theta-burst stimulation or a classical high-frequency stimulation, more in the ventral than the dorsal hippocampus of adult rats (Papaleonidopoulos and Papatheodoropoulos, 2018); however, the same drug concentration has no effect on LTP induced by high-frequency stimulation in either the dorsal or the ventral hippocampus of immature rats (Grigoryan and Segal, 2013). These observations support previous findings that show the dependence of β-AR modulation of LTP on the frequency and the pattern of synaptic activity (Thomas et al., 1996, Gelinas et al., 2008). The picture of β-AR actions along the long axis of the hippocampus becomes even more complex when forms of short-term plasticity are also considered. Thus, 1 μM isoproterenol converts short-term synaptic plasticity into LTP in the dorsal but not ventral hippocampus of immature or adult rats (Grigoryan and Segal, 2013, Grigoryan et al., 2015), presumably acting on a substrate of metaplasticity mechanisms. Furthermore, activation of β-ARs by 1 μM isoproterenol enhances intermitted theta activity bursts more in the ventral than the dorsal hippocampus (Papaleonidopoulos and Papatheodoropoulos, 2018), while the present study shows that β-ARs enhance short-lasting bursts at a relatively wide frequency spectrum (3–40 Hz) in the dorsal hippocampus only. These data suggest that the β-AR modulation of neural activity along the hippocampus depends on several factors, including the specific pattern of presynaptic activity, the age of animals and the potential presence of mechanisms of metaplasticity. Here, it is emphasized that short-term changes in neural activity play different functional roles in brain circuits from those performed by long-term plasticity. Thus, while long-term plasticity is involved mainly in the processes of memory formation (Takeuchi et al., 2014), short-term changes in neuronal activity are thought to be involved in current processing of neural activity, such as filtering, amplification and pattern detection (Abbott and Regehr, 2004, Jackman and Regehr, 2017). It is therefore understood that β-AR could differently regulate forms of short-term and long-term plasticity along the hippocampus.

Interestingly, the present results are compatible with the recently proposed hypothesis of “glutamate amplifies noradrenergic effects” (GANE) (Mather et al., 2016). According to GANE hypothesis, under conditions of arousal, mutually enhancing interactions between glutamatergic and adrenergic transmission can lead to locally amplified neural activity, the “hotspot”, thereby favoring the representations that are associated with the hotspot. Importantly, β-ARs represent a key component for the appearance of a hotspot. By analogy, the dorsal hippocampus local network could be considered as a hotspot in which, as the present experiment shows, the combined increased activation of glutamatergic and noradrenergic transmission leads to amplified local network excitation. Thus, it could be assumed that under conditions of intense arousal, hippocampal output is facilitated favorably from the dorsal segment of the structure, presumably by mechanisms supporting β-AR-dependent localized amplification of neural activity.

In conclusion, the present results show that activation of β-ARs increases baseline and short-term changes of neuronal activity in the CA1 region more in the dorsal than the ventral hippocampus, suggesting that β-AR modulation is significantly involved in dissociating the functional properties of the major output region of the hippocampus along its longitudinal axis.

## Ethical Statement

The authors certify that animal experiments were carried out in accordance with the European Communities Council Directive Guidelines for the care and use of Laboratory animals (2010/63/EU – European Commission). The authors also certify that all animal experiments have been approved by the “Protocol Evaluation Committee” of the Department of Medicine of the University of Patras and the Directorate of Veterinary Services of the Achaia Prefecture of Western Greece Region (reg. number: 187531/626, 26/06/2018). The authors attest that all efforts were made to minimize the number of animals used and their suffering.

## CRediT authorship contribution statement

A. Miliou, V. Papaleonidopoulos and G. Trompoukis performed the experiments and analyzed the data, C. Papatheodoropoulos designed and supervised the study, contributed to data analysis, prepared and wrote the manuscript and acquired the funding.

## Conflicts of Interest

The Authors declare no conflict of interest.
